# Recent Advances in Plant Genetics and Genomics

**DOI:** 10.3390/plants15131947

**Published:** 2026-06-24

**Authors:** Salma Kelany, Qianhao Zhu, Longbiao Guo

**Affiliations:** 1 State Key Laboratory of Rice Biology and Breeding, China National Rice Research Institute, Hangzhou 310006, China; salmakelany93@gmail.com; 2 Commonwealth Scientific and Industrial Research Organization, Agriculture and Food, GPO Box 1700, Canberra, ACT 2601, Australia

## 1. Introduction

Publication of the genome sequences of *Arabidopsis thaliana* in 2000 and of *Oryza sativa* in 2002 marked the beginning of an exciting era in which advances in molecular technology and methodology uncovered the genetic blueprints of hundreds of plant species [1]. The rapid development of high-throughput sequencing technology, particularly third-generation sequencing technology, enables not only affordable resequencing of a large number of plant genomes, but also generates gapless telomere-to-telomere genomes of plant species, most importantly of crops pivotal to humanity. These fundamental genomic resources, together with multi-omics data, greatly facilitate exploration of the genetic diversity of plants, the discovery of genes underlying important agronomic traits of crops, and elucidation of the molecular mechanisms of the genes. The resources and knowledge accumulated are now being translated into real-world impact through the breeding of climate-smart new crop varieties, thereby benefiting global food security [2,3,4].

Building on this foundation, this Special Issue, “Recent Advances in Plant Genetics and Genomics”, continues to report the recent advancements in this field. It compiles nine research articles and two review papers covering diverse research topics on major staple crops and other plants, including the screening of germplasm that is tolerant to biotic or abiotic stress, the characterization of a plant’s response to environmental variables, genome-wide identification of the gene family, genome-wide association studies (GWAS), quantitative trait locus (QTL) mapping, and gene cloning. Next, we briefly summarize these studies with the following aims: (i) understanding transcriptional and post-translational gene regulation, (ii) mining QTLs and genes associated with agronomic traits, and (iii) examining the responses of plant growth and development to environment variables.

## 2. Understanding Transcriptional and Post-Translational Gene Regulation

Both the expression and function of genes are regulated at multiple levels, including transcriptional, post-transcriptional, translational, and epigenetic. Three papers included in this Special Issue exemplified transcriptional gene expression and reviewed protein post-translational modifications in response to abiotic stresses.

Apyrases (NTPDases) hydrolyze NTP and NDP, removing their terminal phosphates and producing NMP. The regulation of extracellular ATP [eATP] by “ecto-apyrase” activities is important for phosphate uptake and diverse growth, along with other developmental processes of plants. Wang et al. [5] generated transgenic Arabidopsis lines, overexpressing either a pea apyrase PS (psNTP9) or a modified PS. DM was designated due to double mutations in a potential calmodulin (CaM)-binding site that overlapped a predicted nuclear localization signal site. Using the transgenics, the authors investigated the binding differences in the two proteins in the promoters of Arabidopsis genes using ChIP-seq. This revealed that mutations in the CaM-binding site of PS changed the interacting sites of PS in the promoters of Arabidopsis genes, and, consequently, the expression profiles of the PS target genes. The PS-specific binding sites in promoters of Arabidopsis genes were verified by electrophoretic mobility shift assays (EMSA); DM showed minimal binding to the PS-specific binding sites [5]. These findings demonstrate the role of PS in regulating gene expression on top of its known enzymatic function.

Retrozymes are a class of non-autonomous plant retrotransposons that contain long terminal repeats (LTRs). These LTRs contain hammerhead ribozymes (HHRs) that facilitate the circularization of the retrozyme RNA to form circular RNA. In order to uncover the transcription start site of *Nicotiana benthamiana retrozyme 1* (*NbRZ1*) and its function, Lezzhov et al. [6] compared transcription of the *GUS* reporter gene driven by the 35S promoter, wild-type *NbRZ1* LTR, or a mutated LTR, in which a point mutation (G to A substitution) was introduced in the *NbRZ1* LTR to prevent the retrozyme from self-cleavage. The *GUS* transcript was detected for all three constructs, but the GUS protein was detected only for the *GUS* driven by the 35S promoter and mutated LTR. These results indicated the promoter activity of *NbRZ1* LTR and untranslatability of the LTR-GUS transcript, likely due to the self-cleavage activity of the wild-type ribozyme. The transcription start site of *NbRZ1* was further mapped to an adenosine residue at position 116 of the *NbRZ1* LTR via 5′-RACE. In addition, *NbRZ1* was shown to encode a protein targeted for proteasomal degradation in plant cells; overexpression of this protein in plants is known to causes severe necrosis [6]. These findings imply a potential mechanism involved in regulating the expression of proteins encoded by *NbRZ1* at transcriptional and post-transcriptional levels.

Protein post-translational modifications (PTMs) can regulate the chemical decoration, structures, and functions of translated proteins. Li et al. [7] summarized the molecular mechanisms of major types of PTMs, including phosphorylation, ubiquitination, SUMOylation, glycosylation, methylation, and acetylation, with a focus on their regulatory roles in plant responses to abiotic stresses. Phosphorylation can activate SUMOylation-HSFA1/HSFA2 to regulate the heat stress signaling pathway. Phosphorylation, ubiquitination, and S-acylation collectively regulate the expression of cold-tolerance-related genes. SnRK2s-phosphorylation regulates drought stress response. Upon exposure to salt stress, the coupling of phosphorylation of SOS pathway-related proteins, ubiquitination, and phospholipid metabolism maintains ion homeostasis, conferring salt tolerance. Additionally, PTMs play a key role in ABA-mediated abiotic stress responses via signal transduction involving PYR/PYL/RCAR receptors, PP2Cs, and SnRK2s. The authors also proposed strategies for how PTMs can be manipulated to breed abiotic-stress-resilient crops [7].

Looking ahead, to fully understand gene function and its contribution to phenotype, it is important to investigate the regulatory mechanisms simultaneously at multiple levels, which has been enabled by rapid progress in multi-omics technologies.

## 3. Mining QTLs and Genes Associated with Agronomic Traits

Six studies within this Special Issue reported results on genetic mapping of quantitative trait loci (QTLs) and genes associated with agronomic traits in different plants.

Yardlong bean (*Vigna unguiculata* ssp. *unguiculata* cv.gr. *sesquipedalis*) is a vegetable variety of cowpea, and is an economic crop of several Southeast Asia countries and China. Pod traits determine appearance and are related to eating and cooking qualities. Thua Ngu is a local yardlong bean cultivar in southern Thailand which has a unique snake-like pod appearance and crispiness. In order to determine the genetic basis of the snake-like pod (SLP) trait, Thepphomwong et al. [8] constructed an F2 segregation population by crossing Thua Ngu with Raya, and sequenced the genomes of the two parents and two bulked DNA samples pooled from progeny with extreme traits (SLP and normal pod). QTL-seq analysis using ∆(SNP-index) identified a major QTL, *qSlp4.1*, for the SLP trait on chromosome 4. Further fine-mapping delimited *qSlp4.1* to a 152.88 Kbp region containing nine genes, and potential candidate genes for the SLP trait were shortlisted based on gene annotation, paving the foundation for revealing the genetic basis underlying the trait [8].

Leaf rolling affects plant architecture and photosynthesis. Leng et al. [9] generated a *rolling leaf2* (*rll2*) mutant from *japonica* variety Wuyunjing27 (WYJ27) via ethylmethanesulfonate (EMS) mutagenesis, and identified the underlying gene *RLL2* based on map-based cloning using an F2 population derived from *rll2* x IR36. *RLL2* encoding a plant-specific calpain-like cysteine proteinase was cloned. *RLL2* is mainly expressed in young shoots, roots, panicles, and spikelets. *RLL2* seems to control leaf rolling by regulating bulliform cells; the size and number of bulliform cells were significantly increased in *rll2* compared to those of the wild type [9]. Manipulating the expression of *RLL2* specifically in leaves may allow us to breed rice cultivars with an ideal plant architecture.

To explore the genetic basis of salt tolerance (ST) in Egyptian rice germplasm, Wang et al. [10] re-sequenced 56 Egyptian rice accessions, subjected them to salt stress to evaluate their salt tolerance using the Standard Evaluation System (SES) scale, and performed genome-wide associations (GWAS) in combination with another 258 rice accessions. The investigation identified a total of 19 ST QTLs, with 17 of them being newly identified. The QTLs included three previously reported salt-tolerance-related genes, including *OsIPK1, OsRPH1/STG5*, and *ONAC063*. Comparative RNA-Seq analysis was carried out using Giza 176 (salt-tolerant) and 9311 (salt-susceptible). Interestingly, *ONAC063* was one of the differentially expressed genes, implying a potential role of the gene in the salt response of Giza 176. The role of *ONAC063* in salt tolerance was further confirmed by the finding of a favorable haplotype (Hap3) in the 258 rice accessions [10]. The results showed the power of an integrated approach in accelerating the discovery of functional genes.

The experiment conducted by Jin et al. [11] aimed to identify root system architecture traits related to drought tolerance in wheat. To this end, the authors developed 262 recombinant inbred lines (RILs) derived from Zhoumai16 × DK171 (drought-tolerant, DT). Based on the high-density SNP genetic map, five QTLs accounting for 6.1% to 18.9% of the phenotypic variances were identified based on root weight, length, area, and number of root tips; two overlapped with previously reported QTLs, and three were novel ones. Markers were developed for each QTL, enabling marker-assisted incorporation of the DT loci in the new wheat cultivars [11].

The white-backed planthopper (WBPH) severely impacts rice production. Roddee et al. [12] conducted a set of experiments aiming to establish effective methods for identifying WBPH-resistant rice varieties. The authors first quantified the feeding intensity of WBPH on ten rice varieties based on honeydew excretion and salivary sheath formation after a 24 h feeding. The authors further evaluated the tolerance of the rice varieties to WBPH feeding using the electrical penetration graph technique, the growth/survival rate of WBPH, and the impact of the morphological (trichome) trait. In addition, changes in hormones and secondary metabolites, as well as the expression of resistance-related genes, in rice following WBPH feeding were investigated. The results led the authors to conclude that the method based on the growth/survival rate of WBPH was the most suitable for screening WBPH-resistant rice varieties [12].

Blast is one of the major biotic threats to rice growth and development. Controlling the occurrence of rice blast will ensure sustainable rice production. On the basis of presenting the impact of rice blast on rice production, food security, and global economy, Cheng et al. [13] reviewed the reported rice genes associated with blast resistance or susceptibility along with the immune mechanisms underlying rice blast resistance. The authors also discussed how the findings can be applied in breeding blast-resistant rice varieties [13]. Overall, this article provides an overview of the research progress on rice blast and breeding of rice varieties resistant to blast.

## 4. Responses of Plant Growth and Development to Environment Variables

Light is one of the most important environmental factors determining plant growth and development, affecting both photosynthesis and photomorphogenesis. Two studies included in this Special Issue exemplified how light quality affects plant growth and development in rice and *Chimonobambusa sichuanensis* (an ornamental shrubby bamboo).

Liang et al. [14] investigated the effects of different red-to-blue light ratios on rice seedlings of Jijing129 under an LGI-660/450 dual-wavelength semiconductor laser system. Rice seedlings treated with three red-to-blue light ratios (50:50, 60:40 or 75:25) were compared to those grown under natural light. It was found that all three treatments significantly increased the root weight and nitrogen content of rice seedlings, and even final grain yield, particularly the 60:40 (R:B) ratio. Compared to the seedlings under natural light, those treated with different R:B laser lights showed altered gene expression, with 101, 1645, and 2247 DEGs identified in the 50:50, 60:40, and 75:25 treatments, respectively. DEGs were found to be mainly involved in pathways related to metabolic processes, nitrogen metabolism, and protein amino acid phosphorylation [14]. While the findings are intriguing, it is of interest how the findings can be implemented in commercial rice production.

In the study by Kong et al. [15], the authors first identified four cryptochrome genes (*CRY*) in *Chimonobambusa sichuanensis* using the *Phyllostachys edulis* CRY genes as queries. The newly found genes were designated as *CsCRY1a*, *CsCRY1b*, *CsCRY2*, and *CsCRY3*. The proteins encoded by the four genes together with 55 CRYs proteins of 10 other species were used in a phylogenetic analysis. These CRYs were divided into three subfamilies, with CsCRY1a and CsCRY1b clustered in subfamily I, CsCRY2 in subfamily II, and CsCRY3 in subfamily III. To understand the response of the four genes to different light qualities and temperatures, their expression levels were compared in *C. sichuanensis* seedlings treated with different light qualities (red, blue, and a mixture of red and blue) or temperatures (−5 °C to 20 °C). It was found that the four genes responded differently to light quality; moreover, *CsCRY2* seemed to have a higher expression level than other three genes, particularly under low temperatures [15].

## 5. Conclusions and Perspectives

This Special Issue, entitled “Recent Advances in Plant Genetics and Genomics”, compiles nine research articles and two review papers. The studies display the scope of the research areas, from the characterization of germplasm in response to abiotic and biotic stress to the identification of potential genes underlying important agronomic traits. The studies also showcase the application of a wide range of tools and methodologies in plant genetics and genomics research. The practical approaches suggested by the review papers not only provide tools for further advancement in the areas of the review papers’ focus, but are also widely applicable to other related areas.

Agriculture—specifically, crop production—is currently facing a series of challenges, such as high temperatures, drought, soil salinization, and biotic stresses, which can lead to crop yields dropping by more than 30%. Combating these challenges necessitates enhancing studies on plant genetics, genomics, and functional genomics. Crop germplasms that have undergone long-term adaptation to extreme environments are invaluable resources and provide a critical foundation for future breakthroughs in crop improvement. Their allelic variation should be systematic explored through the integration of multi-omics approaches, so as to identify and elucidate the complex genetic architectures and regulatory pathways underlying key agronomic traits and stress tolerances. With that, advanced breeding strategies, including marker-assisted selection, CRISPR/Cas9-mediated genome editing, and breeding by design, can then be implemented in breeding crop varieties that combine high yield potential, superior quality, and resilience to diverse environmental stresses [16]. Ultimately, future research on plant genetics, genomics, and functional genomics should follow the conceptual framework of ‘mining elite genes—dissecting genetic mechanisms—developing novel germplasm—implementing in crop breeding’.

## Data Availability

No new data were created or analyzed in this study. Data sharing is not applicable to this article.
